# Artificial intelligence in forensic science: a systematic review. Part I: personal identification

**DOI:** 10.1007/s00414-026-03855-5

**Published:** 2026-06-04

**Authors:** Valentina Bugelli, Francesco Calabrò, Laura Donato, Rossana Cecchi, Jessika Camatti, Marco Di Paolo, Lorenzo Franceschetti

**Affiliations:** 1 University of Parma, Parma, Italy; 2 University of Rome Tor Vergata, Rome, Italy; 3 University of Modena and Reggio Emilia, Modena, Italy; 4 University of Pisa, Pisa, Italy; 5 University of Milano, Milan, Italy

**Keywords:** Artificial intelligence, Forensic identification, Forensic anthropology, Machine learning, Deep learning, Sex estimation

## Abstract

Artificial intelligence (AI) has emerged as a promising tool in forensic sciences, offering new opportunities for personal identification through automated analysis of biological and imaging data. AI-based approaches have been increasingly applied to tasks such as sex estimation, human identification, ancestry estimation, and kinship analysis. This systematic review aims to synthesize the available evidence regarding the applications, methodological characteristics, and performance of AI models in forensic personal identification. A systematic literature search was conducted in PubMed/MEDLINE and Scopus following PRISMA guidelines. Studies investigating AI applications for forensic identification were included. Data extraction focused on study characteristics, dataset type, AI model architecture, forensic task, validation strategy, and reported performance metrics. A total of 89 studies published between 2012 and 2026 met the inclusion criteria. The majority of studies focused on sex estimation (63%), followed by human identification, ancestry estimation, multi-task prediction, and kinship verification. Most studies relied on imaging datasets, particularly computed tomography and radiographic images. Deep learning models represented the most frequently used analytical approaches. Reported accuracy values were generally high, with a median accuracy of 91.4% and an interquartile range of 88.9–95.0% in studies reporting single-value accuracy metrics. Deep learning approaches tended to achieve slightly higher performance than traditional machine learning models. AI shows considerable potential to support forensic personal identification, particularly in imaging-based applications. However, methodological heterogeneity, population-specific datasets, and limited external validation remain important challenges. Future research should prioritize standardized validation protocols, multi-population datasets, and transparent reporting to ensure the forensic applicability of AI-based identification systems.

**Supplementary Information:**

The online version contains supplementary material available at 10.1007/s00414-026-03855-5.

## Introduction

Forensic personal identification represents one of the core objectives of forensic science, particularly in cases involving unidentified human remains, mass disasters, and criminal investigations. Establishing the identity of deceased individuals is essential for legal investigations and humanitarian reasons, including disaster victim identification (DVI) and providing closure for families. The reconstruction of the biological profile—typically including sex, age, ancestry, and stature—plays a fundamental role in the identification process, especially when soft tissues are absent or when bodies are severely decomposed, fragmented, or skeletonized [1].

Traditionally, forensic identification relies on multiple complementary disciplines, including forensic anthropology, forensic odontology, and forensic genetics. Dental structures and craniofacial features are particularly valuable due to their durability and individual variability, making them useful for identifying victims in both criminal investigations and mass disaster scenarios [2]. In parallel, DNA profiling has become the gold standard for human identification, providing highly reliable genetic evidence that can link biological samples to individuals with high statistical confidence [3]. Additionally, morphological and metric analyses of skeletal structures remain fundamental tools in forensic anthropology for estimating biological characteristics when genetic material is unavailable or degraded [4].

In recent years, the rapid development of computational technologies has introduced new possibilities for forensic investigations through the application of artificial intelligence (AI). AI is a broad term describing computational systems capable of performing tasks that typically require human intelligence. Within AI, machine learning (ML) refers to algorithms that learn patterns from data to make predictions or classifications without explicit programming. Deep learning (DL) represents a subset of machine learning based on multi-layer artificial neural networks, particularly effective for analyzing complex imaging and high-dimensional datasets. [5]. These techniques have already demonstrated significant potential in several fields of medicine and science, enabling automated analysis of complex biological, imaging, and genetic data [6].

The integration of AI into forensic sciences is gaining increasing attention, with applications spanning forensic pathology, crime scene analysis, forensic radiology, and human identification [7]. Machine learning algorithms can assist forensic experts by identifying patterns in large datasets, improving classification accuracy, and supporting decision-making processes while potentially reducing subjective bias [8]. In particular, AI-based approaches have been explored in various identification-related domains, including facial reconstruction, skeletal analysis, genetic profiling, and microbiome-based identification [9, 10].

Despite the growing number of studies investigating the application of AI in forensic identification, the available evidence remains dispersed across different forensic disciplines and methodological approaches. Existing studies often focus on specific domains—such as forensic genetics, anthropology, or imaging—without providing a comprehensive synthesis of AI applications across the broader context of forensic personal identification. Therefore, a systematic evaluation of the current literature is needed to better understand the potential, limitations, and methodological challenges associated with the implementation of AI in this field.

Therefore, the aim of this systematic review was to analyze and synthesize the available evidence regarding the application of AI techniques in forensic personal identification.

## Materials and methods

### Study design and research question

This study was designed as a systematic review aimed at evaluating the application of AI techniques in forensic personal identification. The review was conducted and reported in accordance with the Preferred Reporting Items for Systematic Reviews and Meta-Analyses (PRISMA 2020) guidelines [11].

The study protocol was defined a priori, including the research question, eligibility criteria, and data extraction strategy, in order to ensure methodological transparency and reproducibility. The study protocol was prospectively registered on the Open Science Framework (OSF) platform. A publicly accessible view-only version of the protocol is available at: https://osf.io/39phc/overview?view_only=c223168473564131aec86a269e1beacd.

The aim of this review was to investigate the performance, applicability, and limitations of AI–based approaches used for forensic personal identification, including human identification from imaging data, sex estimation, ancestry or population affinity estimation, and facial recognition in forensic contexts.

The research question was structured according to a modified PICO framework adapted for methodological studies in forensic science: Population/Data: forensic imaging, odontological, anthropological, or other biological datasets; Intervention: AI-based analytical methods; Comparator: conventional forensic methods or human expert assessment when available; Outcome: identification performance metrics (e.g., accuracy, sensitivity, specificity, area under the curve (AUC), equal error rate (EER), and rank-based accuracy).

### Eligibility criteria and search strategy

Studies were selected according to the following inclusion criteria: original research articles involving AI-based methods (e.g., machine learning, deep learning, neural networks, or related computational approaches); forensic or medico-legal applications related to personal identification; studies employing imaging, anthropometric, odontological, or other biological data for identification purposes; studies reporting quantitative performance metrics (e.g., accuracy, sensitivity, specificity, AUC, EER, or rank-based accuracy); peer-reviewed articles published in English.

Exclusion criteria were: studies focused exclusively on clinical diagnosis without relevance to forensic identification; editorials, letters, conference abstracts without full data, and opinion papers; studies lacking objective performance metrics. Studies exclusively focused on forensic age estimation were excluded from this review, as this topic is addressed in a separate systematic review within the same three-part series on artificial intelligence applications in forensic science. Conference proceedings and abstracts were excluded due to limited methodological detail and lack of full peer-reviewed publication.

A systematic literature search was conducted in MEDLINE (via PubMed) and Scopus from database inception to the final search date (1 March 2026). The electronic search was supplemented by backward and forward citation tracking in order to identify additional relevant studies. The complete database-specific search strategies are reported in Supplementary Appendix 1. Reference lists of the included studies were also manually screened to identify additional eligible articles.

### Study selection and data extraction

All records retrieved from the databases were exported and imported into Zotero reference management software, where duplicate records were identified and removed. Two independent reviewers performed the screening process in two stages: title and abstract screening; full-text assessment for eligibility. Disagreements between reviewers were resolved through discussion and consensus.

Data extraction was conducted using a standardized data extraction form developed prior to the screening phase. The following information was collected from each included study: author(s), year of publication, and journal; dataset characteristics (sample size, population, and type of data); AI model architecture; task type (e.g., classification, identification, or prediction); reported performance metrics; comparator methods (human experts or conventional forensic techniques, when available); and methodological limitations. Data extraction was independently performed by two reviewers and subsequently cross-checked to ensure accuracy and completeness.

### Risk of bias, methodological quality assessment, and data synthesis

Methodological quality and risk of bias were evaluated using tools appropriate for diagnostic and predictive studies involving AI. Depending on study design, the following frameworks were applied: QUADAS-2 for diagnostic accuracy studies; PROBAST, adapted for AI prediction models. Particular attention was paid to potential sources of bias specific to AI-based studies, including dataset imbalance, lack of external validation, and possible overfitting of predictive models. In addition, adherence to AI reporting standards (e.g., CLAIM or TRIPOD-AI) was qualitatively assessed when applicable.

Given the expected methodological heterogeneity in datasets, model architectures, and outcome metrics, a narrative synthesis was planned as the primary method of analysis. Included studies were grouped according to their main application domain: dental-based human identification; facial recognition in forensic contexts; sex estimation; ancestry or population affinity estimation. When sufficient methodological homogeneity was identified among studies, quantitative comparison of reported performance metrics was considered.

## Results

### Study selection

The literature search identified a total of 1,266 records through database searching, including 396 records from PubMed/MEDLINE and 870 records from Scopus. After removal of 185 duplicate records and one retracted article, 1,080 records remained for title and abstract screening. During the screening phase, 863 records were excluded based on title and abstract because they did not meet the eligibility criteria. The full texts of 217 articles were then sought for retrieval. Of these, 14 reports could not be retrieved, leaving 203 articles assessed for full-text eligibility.

**Fig. 1:**
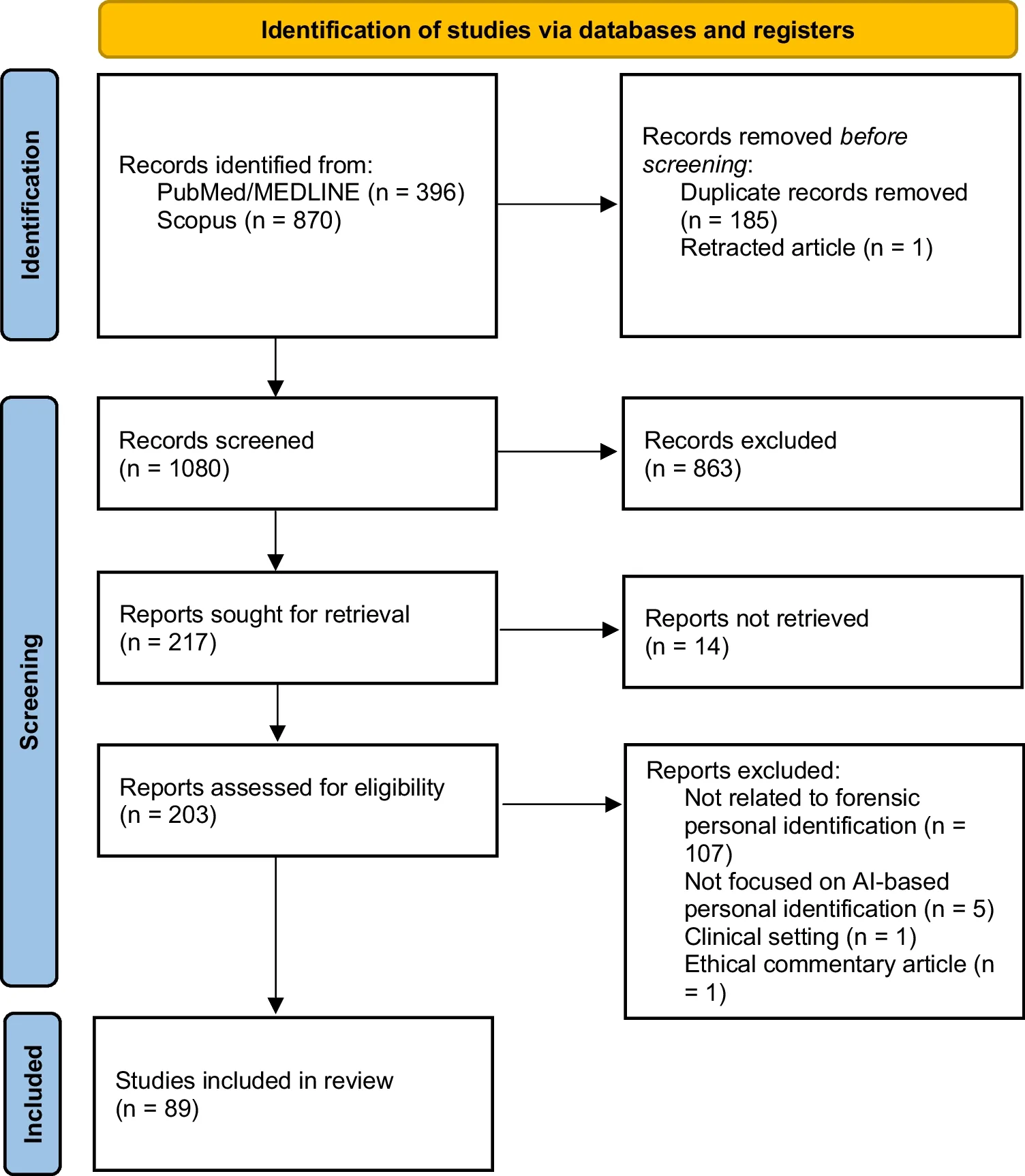
PRISMA Flow diagram of the systematic review

Following full-text evaluation, 114 articles were excluded for the following reasons: not related to forensic personal identification (*n* = 107); not focused on AI-based personal identification (*n* = 5); clinical setting without forensic relevance (*n* = 1); ethical commentary article (*n* = 1). Ultimately, 89 studies met the inclusion criteria and were included in the qualitative synthesis of this systematic review [12–100]. The study selection process is summarized in Fig. 1 (PRISMA flow diagram).

### Characteristics of included studies

The included studies were published between 2012 and 2026 and covered multiple forensic applications of AI, including sex estimation, ancestry estimation, human identification, and kinship verification.

The studies were conducted across several geographic regions. The majority of studies originated from Asia, particularly China, Japan, Turkey, and South Korea, followed by Europe, South America, and North America. Sample sizes varied substantially across studies, ranging from 10 individuals in microbiome-based identification research to over 200,000 radiographic images in large dental datasets.

The main characteristics of the studies included in this systematic review are summarized in Table 1, while a detailed description of each study is provided in Supplementary Table S1.

**Table 1:** Summary characteristics of the studies included in the systematic review (*n* = 89)

Characteristic	Category	Studies n (%)
Forensic task	Sex estimation	56 (63.0)
	Human identification	14 (15.7)
	Ancestry estimation	9 (10.1)
	Multi-task prediction	6 (6.7)
	Kinship verification	4 (4.5)
Data type	Computed tomography-based datasets	34 (38.2)
	Conventional radiographs	21 (23.6)
	Dental panoramic radiographs	10 (11.2)
	Photographic skeletal images	12 (13.5)
	Other datasets	12 (13.5)
AI model category	Deep learning	47 (52.8)
	Traditional machine learning	28 (31.5)
	Hybrid approaches	14 (15.7)

### Data types and imaging modalities

A wide range of anatomical structures and imaging modalities were analyzed across the included studies. The most frequently used data sources included: computed tomography (CT) scans, particularly cranial and pelvic CT datasets; dental panoramic radiographs (orthopantomograms); conventional radiographs (X-ray images); three-dimensional skeletal reconstructions; photographic images of skeletal elements; facial and ear biometric images; genetic and microbiome profiles.

Among imaging-based approaches, CT-derived measurements and radiographic datasets represented the most common data types, reflecting their widespread use in forensic anthropology and forensic identification research.

### AI approaches

A wide variety of AI models were employed across the included studies. The most commonly used algorithms included: Convolutional Neural Networks (CNNs); Artificial Neural Networks (ANNs); Random Forest (RF); Support Vector Machines (SVM); k-Nearest Neighbors (KNN); Logistic Regression (LR); Gradient Boosting and XGBoost models. Deep learning architectures such as ResNet, EfficientNet, GoogLeNet, and VGG-based networks were frequently used in studies involving medical imaging. Several studies also implemented ensemble learning approaches, combining multiple machine learning algorithms to improve predictive performance.

Imaging-based datasets were predominantly analyzed using deep learning architectures, particularly convolutional neural networks, whereas studies relying on morphometric or osteometric measurements more frequently employed traditional machine learning algorithms such as Random Forest or Support Vector Machines.

### Forensic tasks and model performance

The majority of studies focused on sex estimation, which represented the most common forensic application across the literature. Other investigated tasks included human identification, ancestry or population affinity estimation, kinship verification, and multi-task prediction frameworks combining sex and age estimation.

Overall, AI approaches demonstrated high predictive performance across most forensic applications. In sex estimation studies, reported accuracies frequently exceeded 85–90%, with several deep learning models achieving accuracy values above 95%. The highest reported performances were observed in CT-based deep learning studies, particularly in pelvic and cranial analyses, where accuracies approached 100% in some datasets. Similarly, identification systems based on dental radiographs or biometric features showed high rank-based identification accuracy, often exceeding 90%. However, performance varied depending on the anatomical structure analyzed, dataset size, and population characteristics, highlighting the importance of population-specific validation.

**Fig. 2:**
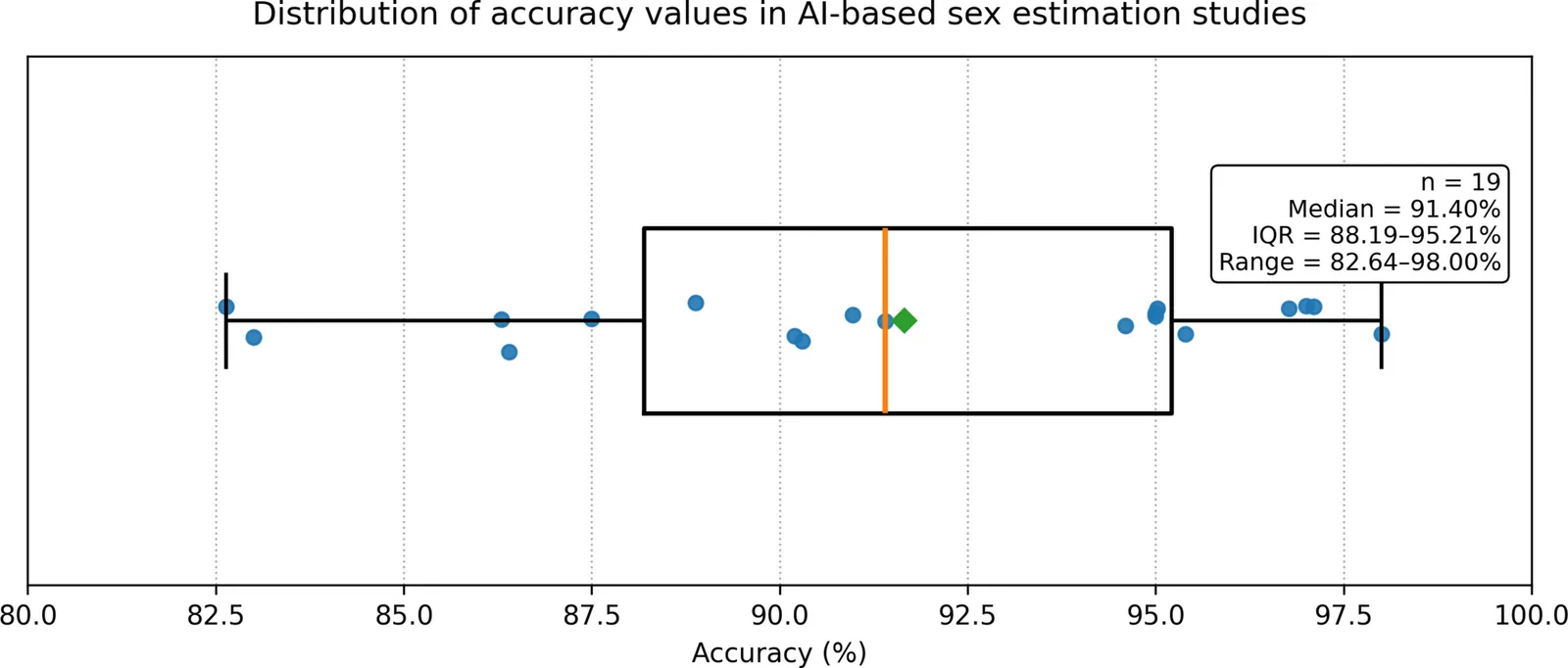
Distribution of accuracy values reported in artificial intelligence models for sex estimation. Boxplot representing the distribution of reported accuracy values across studies providing explicit numeric accuracy outcomes. The central line represents the median, the box indicates the interquartile range (IQR), and whiskers represent the minimum and maximum values. Individual points correspond to accuracy values reported by each included study

**Fig. 3:**
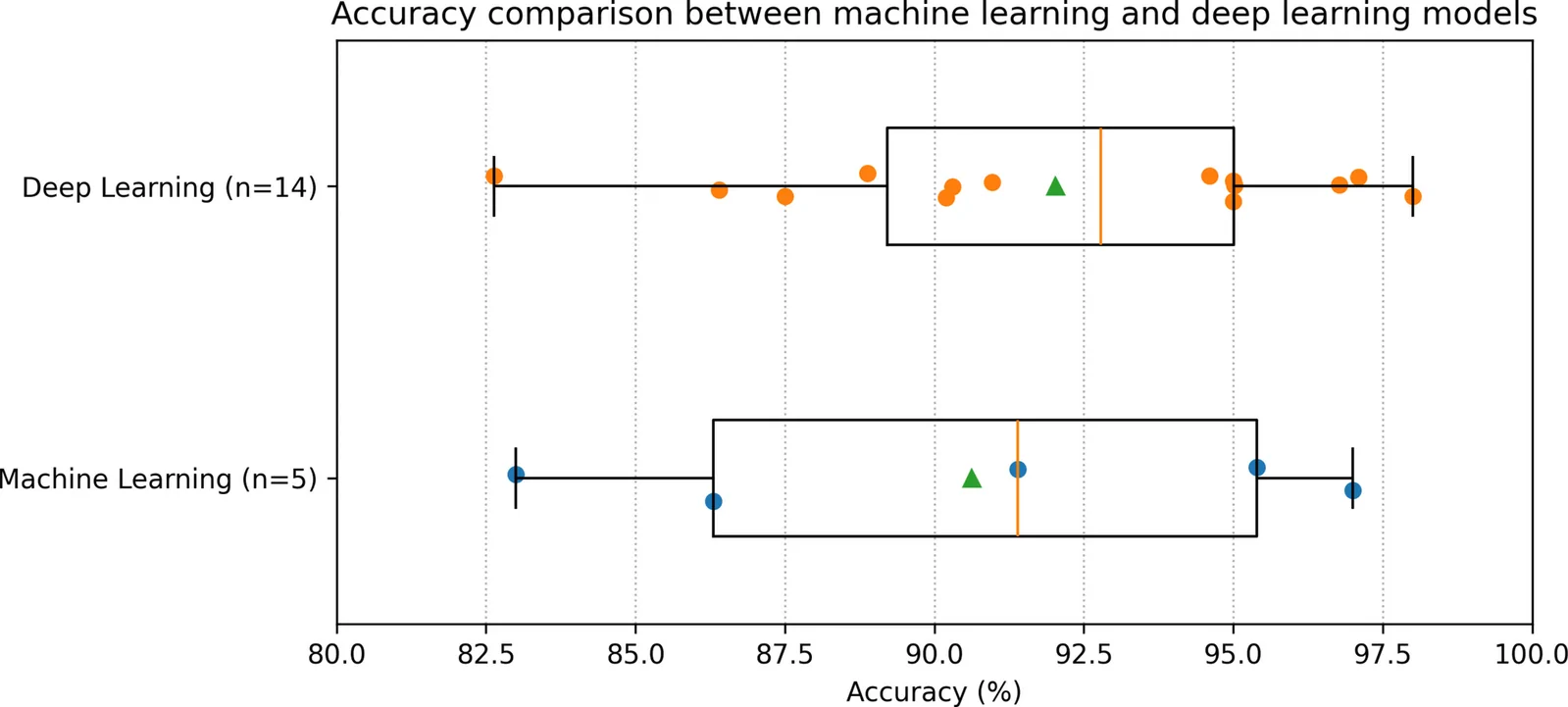
Comparison of accuracy distributions between traditional machine learning and deep learning models used for sex estimation. Boxplots showing the distribution of reported accuracy values across studies using traditional machine learning algorithms (*n* = 5) and deep learning models (*n* = 14). The central line represents the median, boxes indicate the interquartile range (IQR), and whiskers represent the minimum and maximum values. Individual points correspond to accuracy values reported by each included study

Across studies reporting single-value accuracy metrics, the median accuracy was 91.4%, with an interquartile range from 88.9% to 95.0%. The distribution of accuracy values across studies is illustrated in Fig. 2. When stratified by model category, deep learning approaches showed slightly higher median accuracy values than traditional machine learning algorithms, although substantial overlap between distributions was observed (Fig. 3).

The heterogeneity of datasets, evaluation metrics, and methodological approaches limited the possibility of conducting a formal meta-analysis.

### Geographical distribution of studies and population representation

The included studies demonstrated a broad geographical distribution, although a clear concentration of research activity was observed in specific regions. Most studies were conducted in Asia, particularly in China, Turkey, Japan, South Korea, and Thailand, which together accounted for the largest proportion of published datasets. A substantial number of studies also originated from Europe, including populations from France, Portugal, Italy, Greece, Spain, and Bulgaria. Additional contributions were reported from North America, South America, and Africa, although these regions were comparatively less represented.

Several studies used population-specific skeletal collections or hospital-based imaging datasets, often reflecting the demographic composition of the local population. Despite this geographic diversity, the overall distribution of datasets revealed uneven population representation, with a predominance of studies conducted on East Asian and Turkish populations. In contrast, African, South American, and multi-ancestry populations remain underrepresented.

### Temporal trends in AI applications

An increasing trend in the application of AI to forensic anthropology and identification was observed over time. The earliest studies included in this review date back to 2012, when machine learning approaches were primarily applied to biometric identification tasks, such as ear recognition or basic morphometric classification. Between 2015 and 2019, the number of publications gradually increased, with the introduction of more advanced machine learning techniques applied to skeletal morphometrics and radiological datasets. During this period, algorithms such as Support Vector Machines, Random Forest, and Artificial Neural Networks were commonly used. A marked increase in publications was observed after 2020, coinciding with the rapid adoption of deep learning architectures and the growing availability of large medical imaging datasets. Recent studies published between 2023 and 2026 frequently incorporated advanced architectures, including ResNet, EfficientNet, Transformer-based networks, and hybrid deep learning frameworks, often achieving higher predictive performance than traditional machine learning approaches.

Overall, these trends indicate a clear shift from traditional morphometric statistical models toward data-driven AI methods, reflecting broader developments in medical imaging and computational anthropology.

## Discussion

This systematic review analyzed the current evidence regarding the application of AI techniques in forensic personal identification. A total of 89 studies were included, covering a wide range of forensic tasks such as sex estimation, human identification, ancestry or population affinity estimation, and kinship verification [12–100]. Overall, the findings of this review indicate that AI-based approaches demonstrate high predictive performance across multiple forensic applications. In studies reporting single accuracy metrics, the median accuracy was approximately 91%, suggesting that AI methods may provide valuable support for forensic identification tasks. Sex estimation represented the most frequently investigated application, while deep learning approaches—particularly convolutional neural networks applied to radiological and photographic datasets—were the most commonly used model architectures. Despite these promising results, substantial heterogeneity was observed across datasets, methodological approaches, and evaluation metrics, highlighting the need for careful interpretation of reported performances.

One of the most consistent findings across the included studies was the high predictive performance achieved by AI models in sex estimation tasks. Many studies reported accuracy values exceeding 85–90%, with several deep learning models achieving performance above 95%, consistent with the overall accuracy distribution observed in the included studies (Fig. 2) and with individual studies reporting very high performance in CT-based skeletal analyses [56, 81, 92]. These results are consistent with the well-established sexual dimorphism of specific skeletal regions—particularly the pelvis and skull—which are widely used in forensic anthropology for biological sex estimation [2, 4, 101]. These anatomical differences provide biologically informative features that can be effectively captured by machine learning algorithms. AI-based approaches may therefore enhance traditional morphometric analyses by automatically identifying complex patterns within multidimensional datasets and by reducing potential observer-related variability.

In addition to metric and morphometric approaches, individual skeletal features and pathological variations may also contribute to forensic identification and anthropological reconstruction. Case-based investigations and historical osteological studies have demonstrated how specific skeletal traits, trauma patterns, or anatomical anomalies can assist in reconstructing biological profiles and identifying human remains in both modern forensic contexts and historical investigations [102–104]. These traditional anthropological approaches remain essential in forensic practice and provide valuable reference frameworks for the development and validation of AI-based analytical models.

When comparing model categories, deep learning approaches tended to achieve slightly higher accuracy values than traditional machine learning algorithms. This trend was particularly evident in studies using imaging datasets, where convolutional neural networks demonstrated strong performance in tasks involving radiographs, computed tomography scans, and photographic skeletal images [25, 41, 92]. The ability of deep learning models to automatically extract hierarchical visual features from high-dimensional image data likely contributes to their improved performance in these contexts. However, the difference between deep learning and traditional machine learning models was not always substantial. In several studies relying on structured morphometric measurements, algorithms such as Random Forest, Support Vector Machines, or ensemble learning approaches performed comparably to deep learning models [14, 34, 47]. These findings suggest that the optimal modeling strategy may depend largely on the type and structure of the available data.

Another important observation emerging from this review is the prominent role of medical imaging datasets in AI-based forensic identification research. Computed tomography scans, conventional radiographs, and dental panoramic radiographs represented the most commonly used data sources across the included studies. The increasing availability of digital medical imaging, particularly post-mortem computed tomography (PMCT) in forensic practice, has likely facilitated the rapid adoption of AI-based analytical approaches [5, 7]. Imaging-based datasets offer several advantages, including high anatomical detail, standardized acquisition protocols, and the possibility of extracting large numbers of quantitative features from skeletal structures. As a result, AI-driven image analysis may represent one of the most promising directions for future developments in forensic identification.

Imaging-based approaches have long played a central role in forensic identification, particularly in facial comparison and age progression techniques used in missing persons investigations. These methods rely on the analysis of craniofacial morphology and growth patterns and have been extensively discussed in forensic medicine literature [105–113].

Despite the encouraging performance of AI models reported in the literature, several methodological and practical challenges remain. One of the most notable issues concerns the geographical and demographic distribution of the datasets used for model development. A large proportion of the included studies were conducted on Asian populations, particularly in China, Turkey, Japan, and South Korea. European populations were also represented in several studies, whereas African and South American populations were considerably underrepresented. This imbalance raises important concerns regarding the generalizability of AI models across different populations. Skeletal morphology and biological traits may vary between populations due to genetic, environmental, and developmental factors [19, 49]. Consequently, models trained on population-specific datasets may not perform equally well when applied to individuals from different demographic backgrounds. Addressing this limitation will require the development of more diverse and multi-population datasets in future research.

Another challenge identified in this review relates to methodological heterogeneity across studies. The included studies differed substantially in terms of dataset size, anatomical structures analyzed, model architectures, and evaluation metrics. Sample sizes ranged from fewer than ten individuals in microbiome-based identification studies to more than two hundred thousand radiographic images in large dental datasets. Similarly, performance was reported using a variety of metrics, including accuracy, AUC, sensitivity, specificity, equal error rate, and rank-based identification measures. This heterogeneity limited the possibility of conducting a formal meta-analysis and complicates direct comparisons between studies. Standardization of reporting practices and evaluation metrics would therefore greatly facilitate future evidence synthesis in this rapidly evolving field.

In addition to dataset heterogeneity, several studies exhibited methodological limitations that are commonly encountered in AI-based predictive modeling. These include small training datasets, potential class imbalance, limited transparency regarding model development, and the absence of external validation. External validation using independent datasets is particularly important to assess the robustness and real-world applicability of predictive models. However, many studies relied exclusively on internal validation procedures such as cross-validation or hold-out testing within the same dataset. Without independent validation, reported performance metrics may overestimate the true predictive ability of the models when applied in forensic casework.

The findings of this review are broadly consistent with previous research highlighting the growing role of AI in forensic sciences [5–8]. Recent reviews in forensic medicine and forensic radiology have similarly emphasized the potential of AI-based approaches to assist experts in tasks such as skeletal analysis, dental comparison, and biometric identification. In this context, AI should not be considered a replacement for forensic expertise, but rather a complementary tool capable of supporting expert decision-making and improving analytical efficiency. Integrating AI-based systems with expert interpretation may ultimately enhance both accuracy and reproducibility in forensic identification processes.

The integration of AI into forensic identification also raises important ethical and legal considerations. In medico-legal contexts, algorithmic outputs may influence judicial decisions, making transparency, interpretability, and methodological robustness essential requirements for AI-based systems [5–8]. The integration of complex “black-box” models may limit the ability of forensic experts to explain how specific predictions are generated, potentially creating challenges in legal settings where expert testimony must be clearly justified [114]. In addition, the use of population-specific datasets may introduce potential biases if models are applied to individuals from underrepresented demographic groups [5–8]. Ensuring transparency in model development, rigorous validation procedures, and adherence to emerging reporting guidelines for AI research will therefore be crucial for the responsible implementation of AI in forensic practice.

This review has several strengths. First, it provides a comprehensive synthesis of the available literature on AI-based forensic personal identification across multiple forensic disciplines, including anthropology, odontology, radiology, and biometrics. Second, the review was conducted in accordance with PRISMA guidelines and followed a predefined protocol registered on the Open Science Framework, which increases methodological transparency and reproducibility. Finally, the inclusion of a large number of studies allowed the identification of broad methodological trends and research patterns within the field.

Nevertheless, several limitations should be acknowledged. The review included only peer-reviewed studies published in English, which may introduce a degree of publication bias. In addition, the substantial heterogeneity in datasets, model architectures, and performance metrics prevented quantitative meta-analysis of the results. Finally, the rapidly evolving nature of AI research means that new methodological developments may emerge quickly after the completion of the literature search.

Future research should focus on several key priorities. The development of larger and more diverse datasets, including multi-population skeletal and radiological collections, will be essential to improve the generalizability of AI models. External validation using independent datasets should become a standard practice in the evaluation of forensic AI systems. Furthermore, the integration of explainable AI techniques may help improve transparency and interpretability, which are critical factors in medico-legal contexts where algorithmic decisions may be subject to legal scrutiny. Ultimately, interdisciplinary collaboration between forensic scientists, data scientists, and clinicians will be crucial to ensure the responsible and effective implementation of AI technologies in forensic identification.

## Conclusions

This systematic review provides a comprehensive overview of the current applications of AI in forensic personal identification. The findings indicate that AI-based approaches, particularly deep learning models applied to imaging datasets, have demonstrated high predictive performance across several forensic tasks, including sex estimation, human identification, ancestry estimation, and kinship analysis. The increasing availability of digital imaging data, such as computed tomography and radiographic datasets, has played a key role in facilitating the development of AI-driven analytical methods in forensic research.

Despite these promising results, important challenges remain. The predominance of population-specific datasets, methodological heterogeneity across studies, and the limited availability of externally validated models highlight the need for more standardized research protocols and multi-population datasets. In addition, ethical and legal considerations related to transparency, interpretability, and the potential forensic use of algorithmic outputs must be carefully addressed before widespread implementation in medico-legal practice.

Overall, AI should be considered a complementary tool capable of supporting forensic experts rather than replacing human expertise. Future research should focus on improving model transparency, expanding population diversity in training datasets, and developing standardized validation frameworks to ensure the reliability and forensic applicability of AI-based identification systems.

## Supplementary Information

Below is the link to the electronic supplementary material.

Supplementary file1 Supplementary Appendix 1: Database search strategies. Supplementary Table 1: Characteristics of the studies included in the systematic review. (DOCX 46 KB)

## Data Availability

This study is a systematic review of previously published literature. No new datasets were generated during the current study. All data underlying the findings of this review are available in the published articles included in the review and in the supplementary materials provided by the respective authors.

## References

[1] da Silva JC, Strazzi-Sahyon HB, Andreo JC et al (2023) A systematic review of photogrammetry as a reliable methodology in gender identification of human skull. J Forensic Leg Med 97:102546. 10.1016/j.jflm.2023.102546 37307776 10.1016/j.jflm.2023.102546

[2] Jayakrishnan JM, Reddy J, Vinod Kumar RB (2021) Role of forensic odontology and anthropology in the identification of human remains. J Oral Maxillofac Pathol 25:543–547. 10.4103/jomfp.jomfp_81_21 35281159 10.4103/jomfp.jomfp_81_21 PMC8859612

[3] Panneerchelvam S, Norazmi MN (2023) DNA profiling in human identification: from past to present. Malays J Med Sci 30:5–21. 10.21315/mjms2023.30.6.2 38239252 10.21315/mjms2023.30.6.2 PMC10793127

[4] Guleria A, Krishan K, Sharma V, Kanchan T (2023) Methods of forensic facial reconstruction and human identification: Historical background, significance, and limitations. Naturwissenschaften 110:8. 10.1007/s00114-023-01838-9 36807002 10.1007/s00114-023-01838-9

[5] Volonnino G, De Paola L, Spadazzi F (2024) Artificial intelligence and future perspectives in forensic medicine: a systematic review. Clin Ter 175:193–202. 10.7417/CT.2024.5062 38767078 10.7417/CT.2024.5062

[6] Vodanović M, Subašić M, Milošević DP, Galić I, Brkić H (2023) Artificial intelligence in forensic medicine and forensic dentistry. J Forensic Odontostomatol 41:30–41 37634174 PMC10473456

[7] Sacco MA, Tarzia P, Tarda L et al (2024) The artificial intelligence in autopsy and crime scene analysis. Clin Ter 175:192–195. 10.7417/CT.2024.5114 39101424 10.7417/CT.2024.5114

[8] Szeremeta M, Janica J, Niemcunowicz-Janica A (2024) Artificial intelligence in forensic medicine and related sciences - selected issues. Arch Med Sadowej Kryminol 74:64–76. 10.4467/16891716AMSIK.24.005.19650 39450596 10.4467/16891716AMSIK.24.005.19650

[9] Barash M, McNevin D, Fedorenko V, Giverts P (2024) Machine learning applications in forensic DNA profiling: A critical review. Forensic Sci Int Genet 69:102994. 10.1016/j.fsigen.2023.102994 38086200 10.1016/j.fsigen.2023.102994

[10] Franceschetti L, Lodetti G, Blandino A et al (2024) Exploring the role of the human microbiome in forensic identification: Opportunities and challenges. Int J Legal Med 138:1891–1905. 10.1007/s00414-024-03217-z 38594499 10.1007/s00414-024-03217-z PMC11306296

[11] Page MJ, McKenzie JE, Bossuyt PM et al (2021) The PRISMA 2020 statement: An updated guideline for reporting systematic reviews. BMJ 372:n71. 10.1136/bmj.n71 33782057 10.1136/bmj.n71 PMC8005924

[12] Zhang Y, Cui S, Li J et al (2025) Sex inference based on convolutional neural network analysis of fingerprint data. Sci Rep 15:42872. 10.1038/s41598-025-27114-6 41326454 10.1038/s41598-025-27114-6 PMC12669623

[13] Wen H, Wu W, Fan F et al (2022) Human identification performed with skull’s sphenoid sinus based on deep learning. Int J Legal Med 136:1067–1074. 10.1007/s00414-021-02761-2 35022840 10.1007/s00414-021-02761-2

[14] Warrier V, San-Millán M (2025) A statistical evaluation of the sexual dimorphism of the acetabulum in an Iberian population. Int J Legal Med 139:393–409. 10.1007/s00414-024-03334-9 39327330 10.1007/s00414-024-03334-9 PMC11732899

[15] Wang X, Zhao Z, Yang Y et al (2026) Ear biometrics in forensic identification: From ear similarity quantification to kinship verification driven by deep learning approaches. Int J Legal Med 140:477–488. 10.1007/s00414-025-03636-6 41091208 10.1007/s00414-025-03636-6

[16] Verma R, Bhardwaj N, Singh PD, Bhavsar A, Sharma V (2021) Estimation of sex through morphometric landmark indices in facial images with strength of evidence in logistic regression analysis. Forensic Sci Int Rep 4:100226. 10.1016/j.fsir.2021.100226

[17] Tyagi A, Tiwari P, Bhardwaj P, Chawla H (2021) Prognosis of sexual dimorphism with unfused hyoid bone: artificial intelligence informed decision making with discriminant analysis. Sci Justice 61:789–796. 10.1016/j.scijus.2021.10.002 34802653 10.1016/j.scijus.2021.10.002

[18] Triantafyllou G, Botis GG, Piagkou M et al (2024) Sex estimation through orbital measurements: a machine learning approach for forensic science. Diagnostics Basel 14:2773. 10.3390/diagnostics14242773 39767134 10.3390/diagnostics14242773 PMC11674343

[19] Torimitsu S, Nakazawa A, Flavel A et al (2025) Estimation of population affinity using cranial measurements acquired in multidetector computed tomography images of Japanese and Malay individuals. Int J Legal Med 139:863–873. 10.1007/s00414-024-03386-x 39627577 10.1007/s00414-024-03386-x PMC11850424

[20] Torimitsu S, Nakazawa A, Flavel A et al (2024) Estimation of population affinity using proximal femoral measurements based on computed tomographic images in the Japanese and Western Australian populations. Int J Legal Med 138:2169–2179. 10.1007/s00414-024-03257-5 38763925 10.1007/s00414-024-03257-5 PMC11306720

[21] Torimitsu S, Nakazawa A, Flavel A et al (2024) Population affinity estimation using pelvic measurements based on computed tomographic data acquired from Japanese and Western Australian populations. Int J Legal Med 138:1381–1390. 10.1007/s00414-024-03178-3 38316656 10.1007/s00414-024-03178-3 PMC11164820

[22] Torimitsu S, Nakazawa A, Flavel A et al (2024) Estimation of ancestry from cranial measurements based on MDCT data acquired in a Japanese and Western Australian population. Int J Legal Med 138:1193–1203. 10.1007/s00414-024-03159-6 38252284 10.1007/s00414-024-03159-6 PMC11003893

[23] Toneva DH, Nikolova SY, Agre GP et al (2020) Data mining for sex estimation based on cranial measurements. Forensic Sci Int 315:110441. 10.1016/j.forsciint.2020.110441 32781389 10.1016/j.forsciint.2020.110441

[24] Spiros MC, Hefner JT (2020) Ancestry estimation using cranial and postcranial macromorphoscopic traits. J Forensic Sci 65:921–929. 10.1111/1556-4029.14231 31725188 10.1111/1556-4029.14231

[25] Sonmez S, Ozgen MN, Depreli A et al (2026) Triticeal cartilage in forensic anthropological investigations: sex and stature estimation with a machine learning approach. Int J Legal Med 140:1009–1017. 10.1007/s00414-025-03679-9 41317292 10.1007/s00414-025-03679-9

[26] Sonmez S, Nasip OF, Depreli A et al (2026) Sex estimation from human calvarial bone photographs with deep learning approach. J Forensic Leg Med 118:103070. 10.1016/j.jflm.2026.103070 41485422 10.1016/j.jflm.2026.103070

[27] Siino V, Sears C (2020) Artificially intelligent scoring and classification engine for forensic identification. Forensic Sci Int Genet 44:102162. 10.1016/j.fsigen.2019.102162 31604203 10.1016/j.fsigen.2019.102162

[28] Schroder AGD, Rotta JMF, da Silva RD et al (2025) Sex estimation using machine learning algorithms and morphometric assessment of the sella turcica related parameters. Forensic Imaging 43:200654. 10.1016/j.fri.2025.200654

[29] Sasani H, Etli Y, Tastekin B et al (2024) Sex estimation from measurements of the mastoid triangle and volume of the mastoid air cell system using classical and machine learning methods: a comparative analysis. Am J Forensic Med Pathol 45:51–62. 10.1097/PAF.0000000000000890 38039501 10.1097/PAF.0000000000000890

[30] Santos Mota MJ, Alves Vieira AC, Lima L et al (2025) Enhancing sex determination in forensic anthropology: a comparative analysis of cranial measurements using artificial neural network. Forensic Sci Int Rep 12:100422. 10.1016/j.fsir.2025.100422

[31] Sanil G, Prakasha K, Prabhu S et al (2023) 2D–3D facial image analysis for identification of facial features using machine learning algorithms with hyper-parameter optimization for forensics applications. IEEE Access 11:82521–82538. 10.1109/ACCESS.2023.3298443

[32] Salmanipour A, Memarian A, Tofighi S et al (2023) Prediction of sex, based on skull CT scan measurements in Iranian ethnicity by machine learning-based model. Forensic Imaging 33:200549. 10.1016/j.fri.2023.200549

[33] Sagingalieva A, Lusnig L, Cavalli F, Melnikov A (2025) Hybrid quantum neural networks for computer-aided sex diagnosis in forensic and physical anthropology. Inform Med Unlocked 58:101682. 10.1016/j.imu.2025.101682

[34] Ray A, Ray G, Kürtül İ, Şenol GT (2025) Sex estimation from the variables of talocrural joint by using machine learning algorithms. J Forensic Leg Med 114:102912. 10.1016/j.jflm.2025.102912 40499485 10.1016/j.jflm.2025.102912

[35] Porto LF, Lima LNC, Franco A et al (2020) Estimating sex and age from a face: a forensic approach using machine learning based on photo-anthropometric indexes of the Brazilian population. Int J Legal Med 134:2239–2259. 10.1007/s00414-020-02346-5 32820357 10.1007/s00414-020-02346-5

[36] Pichetpan K, Singsuwan P, Intasuwan P et al (2024) Sex determination using the clavicle by deep learning in a Thai population. Med Sci Law 64:8–14. 10.1177/00258024231169233 37063071 10.1177/00258024231169233

[37] Pereira CP, Correia M, Augusto D et al (2025) Forensic sex classification by convolutional neural network approach by VGG16 model: accuracy, precision and sensitivity. Int J Legal Med 139:1381–1393. 10.1007/s00414-025-03416-2 39853362 10.1007/s00414-025-03416-2

[38] Parlak ME, Etli Y, Beyhan M et al (2025) Sex estimation with ensemble learning: an analysis using anthropometric measurements of piriform aperture. Egypt J Forensic Sci 15:26. 10.1186/s41935-025-00426-4

[39] Park SJ, Yang S, Kim JM et al (2024) Automatic and robust estimation of sex and chronological age from panoramic radiographs using a multi-task deep learning network: a study on a South Korean population. Int J Legal Med 138:1741–1757. 10.1007/s00414-024-03204-4 38467754 10.1007/s00414-024-03204-4 PMC11164743

[40] Palmela Pereira C, Carvalho R, Augusto D et al (2025) Development of artificial intelligence-based algorithms for the process of human identification through dental evidence. Int J Legal Med 139:1835–1850. 10.1007/s00414-025-03453-x 40047854 10.1007/s00414-025-03453-x

[41] Oura P, Korpinen N, Machnicki AL, Junno J-A (2023) Deep learning in sex estimation from a peripheral quantitative computed tomography scan of the fourth lumbar vertebra-a proof-of-concept study. Forensic Sci Med Pathol 19:534–540. 10.1007/s12024-023-00586-6 36773213 10.1007/s12024-023-00586-6 PMC10752832

[42] Oura P, Junno J-A, Hunt D, Lehenkari P, Tuukkanen J, Maijanen H (2023) Deep learning in sex estimation from knee radiographs - a proof-of-concept study utilizing the Terry Anatomical Collection. Leg Med (Tokyo) 61:102211. 10.1016/j.legalmed.2023.102211 36738551 10.1016/j.legalmed.2023.102211

[43] Ortega RF, Irurita J, Campo EJE, Mesejo P (2021) Analysis of the performance of machine learning and deep learning methods for sex estimation of infant individuals from the analysis of 2D images of the ilium. Int J Legal Med 135:2659–2666. 10.1007/s00414-021-02660-6 34269895 10.1007/s00414-021-02660-6

[44] Oner Z, Turan MK, Oner S, Secgin Y, Sahin B (2019) Sex estimation using sternum part lengths by means of artificial neural networks. Forensic Sci Int 301:6–11. 10.1016/j.forsciint.2019.05.011 31128410 10.1016/j.forsciint.2019.05.011

[45] Oliva G, Pinchi V, Bianchi I et al (2022) Three-dimensional dental analysis for sex estimation in the Italian population: a pilot study based on a geometric morphometric and artificial neural network approach. Healthcare 10:9. 10.3390/healthcare10010009 10.3390/healthcare10010009 PMC8775125 35052173

[46] Nonthasaen P, Mahikul W, Chobpenthai T, Achararit P (2023) Sex estimation from Thai hand radiographs using convolutional neural networks. Forensic Sci Int Rep 8:100332. 10.1016/j.fsir.2023.100332

[47] Nogueira L, Santos F, Castier F et al (2023) Sex assessment using the radius bone in a French sample when applying various statistical models. Int J Legal Med 137:925–934. 10.1007/s00414-023-02981-8 36826526 10.1007/s00414-023-02981-8

[48] Navega D, Vicente R, Vieira DN, Ross AH, Cunha E (2015) Sex estimation from the tarsal bones in a Portuguese sample: a machine learning approach. Int J Legal Med 129:651–659. 10.1007/s00414-014-1070-5 25186617 10.1007/s00414-014-1070-5

[49] Navega D, Coelho C, Vicente R et al (2015) AncesTrees: Ancestry estimation with randomized decision trees. Int J Legal Med 129:1145–1153. 10.1007/s00414-014-1050-9 25053239 10.1007/s00414-014-1050-9

[50] Musilová B, Dupej J, Brůžek J et al (2019) Sex and ancestry related differences between two Central European populations determined using exocranial meshes. Forensic Sci Int 297:364–369. 10.1016/j.forsciint.2019.02.034 30879959 10.1016/j.forsciint.2019.02.034

[51] Monum T, Makino Y, Yajima D et al (2024) Sex estimation from the first and second ribs using 3D postmortem CT images in a Japanese population: A comparison of discriminant analysis and machine learning techniques. Forensic Sci Int Rep 10:100386. 10.1016/j.fsir.2024.100386

[52] Malatong Y, Intasuwan P, Palee P et al (2023) Deep learning and morphometric approach for sex determination of the lumbar vertebrae in a Thai population. Med Sci Law 63:14–21. 10.1177/00258024221089073 35306907 10.1177/00258024221089073

[53] Maier CA, Zhang K, Manhein MH, Li X (2015) Palate shape and depth: a shape-matching and machine learning method for estimating ancestry from human skeletal remains. J Forensic Sci 60:1129–1134. 10.1111/1556-4029.12812 26258641 10.1111/1556-4029.12812

[54] Machado CR, Curi JP, da Costa Moraes CA et al (2024) Exploratory analysis of new craniometric measures for the investigation of biological sex using open-access statistical and machine-learning tools on a cone-beam computed tomography sample. Int J Legal Med 138:2595–2605. 10.1007/s00414-024-03259-3 38856752 10.1007/s00414-024-03259-3

[55] Li Z, Xie L, Wang G (2023) Deep learning features in facial identification and the likelihood ratio bound. Forensic Sci Int 344:111576. 10.1016/j.forsciint.2023.111576 36758339 10.1016/j.forsciint.2023.111576

[56] Li Y, Niu C, Wang J et al (2022) A fully automated sex estimation for proximal femur X-ray images through deep learning detection and classification. Leg Med (Tokyo) 57:102056. 10.1016/j.legalmed.2022.102056 35430525 10.1016/j.legalmed.2022.102056

[57] Kuha A, Ackermann J, Junno J-A, Oettlé A, Oura P (2024) Deep learning in sex estimation from photographed human mandible using the Human Osteological Research Collection. Leg Med (Tokyo) 70:102476. 10.1016/j.legalmed.2024.102476 38964075 10.1016/j.legalmed.2024.102476

[58] Kocamış OG, Çiçekcibaşı AE, Açar G et al (2025) The role of the sacroiliac joint in sex estimation: analysis of morphometry and variation types using machine learning techniques. Leg Med (Tokyo) 75:102630. 10.1016/j.legalmed.2025.102630 40349523 10.1016/j.legalmed.2025.102630

[59] Knecht S, Santos F, Ardagna Y et al (2023) Sex estimation from long bones: a machine learning approach. Int J Legal Med 137:1887–1895. 10.1007/s00414-023-03072-4 37526736 10.1007/s00414-023-03072-4

[60] Knecht S, Nogueira L, Servant M et al (2021) Sex estimation from the greater sciatic notch: a comparison of classical statistical models and machine learning algorithms. Int J Legal Med 135:2603–2613. 10.1007/s00414-021-02700-1 34554326 10.1007/s00414-021-02700-1

[61] Knecht S, Morandini P, Biehler-Gomez L et al (2026) Interpretable machine learning for individualized sex estimation from long bones. Int J Legal Med 140:983–995. 10.1007/s00414-025-03635-7 41136625 10.1007/s00414-025-03635-7

[62] Knecht S, Morandini P, Biehler-Gomez L et al (2025) Sex estimation from patellar measurements in a contemporary Italian population: a machine learning approach. Int J Legal Med 139:1371–1380. 10.1007/s00414-024-03359-0 39495285 10.1007/s00414-024-03359-0

[63] Knecht S, Krüger G, Liebenberg L et al (2026) Case-specific accuracy in sex estimation from long bones in forensic anthropology: an “Accuracy x-Factors” approach. Forensic Sci Int 380:112820. 10.1016/j.forsciint.2026.112820 41546940 10.1016/j.forsciint.2026.112820

[64] Kim Y, Yoon Y, Matsunobu Y et al (2024) Gray-scale extraction of bone features from chest radiographs based on deep learning technique for personal identification and classification in forensic medicine. Diagnostics 14:1778. 10.3390/diagnostics14161778 10.3390/diagnostics14161778 PMC11353895 39202266

[65] Kim Y, Usumoto Y, Heo J-H et al (2025) Application of deep learning for detecting implants in computed tomography scout images with multi-institution and multi-vendor for personal identification. Sci Justice 65:101315. 10.1016/j.scijus.2025.101315 40930679 10.1016/j.scijus.2025.101315

[66] Kim I, Yang S, Choi Y et al (2025) Automated sex and age estimation from orthopantomograms using deep learning: a comparison with human predictions. Forensic Sci Int 374:112531. 10.1016/j.forsciint.2025.112531 40544576 10.1016/j.forsciint.2025.112531

[67] Kartal E, Etli Y, Asirdizer M et al (2022) Sex estimation using foramen magnum measurements, discriminant analyses and artificial neural networks on an Eastern Turkish population sample. Leg Med Tokyo 59:102143. 10.1016/j.legalmed.2022.102143 36084487 10.1016/j.legalmed.2022.102143

[68] Karim KT, Mohammad DA, Baban MTA (2026) Sex estimation based on odontometry of maxillary anterior teeth and arch dimensions predicted by machine learning algorithms. J Forensic Sci 71:1224–1234. 10.1111/1556-4029.70282 41656750 10.1111/1556-4029.70282

[69] Intasuwan P, Palee P, Sinthubua A, Mahakkanukrauh P (2022) Comparison of sex determination using three methods applied to the greater sciatic notch of os coxae in a Thai population: dry bone morphology, 2-dimensional photograph morphometry, and deep learning artificial neural network. Med Sci Law 62:261–268. 10.1177/00258024221079092 35139683 10.1177/00258024221079092

[70] Imaizumi K, Usui S, Nagata T, Hayakawa H, Shiotani S (2025) Sex-estimation method for three-dimensional shapes of the skull and skull parts using machine learning. Forensic Sci Int 373:112532. 10.1016/j.forsciint.2025.112532 40527270 10.1016/j.forsciint.2025.112532

[71] Imaizumi K, Bermejo E, Taniguchi K et al (2020) Development of a sex estimation method for skulls using machine learning on three-dimensional shapes of skulls and skull parts. Forensic Imaging 22:200393. 10.1016/j.fri.2020.200393

[72] Huang L, Du J, Ye L et al (2025) Species level and SNP profiling of skin microbiome improve the specificity in identifying forensic fluid and individual. Forensic Sci Int Genet 78:103256. 10.1016/j.fsigen.2025.103256 40073753 10.1016/j.fsigen.2025.103256

[73] Attia MH, Farghaly YT, Abulnoor BAE-S, Curate F (2022) Performance of the supervised learning algorithms in sex estimation of the proximal femur: a comparative study in contemporary Egyptian and Turkish samples. Sci Justice 62:288–309. 10.1016/j.scijus.2022.03.003 35598923 10.1016/j.scijus.2022.03.003

[74] Guo Y-X, Lan J-L, Song Y-X et al (2024) Different machine learning methods based on maxillary sinus in sex estimation for Northwestern Chinese Han population. Int J Legal Med 138:2147–2155. 10.1007/s00414-024-03255-7 38760564 10.1007/s00414-024-03255-7

[75] Gomez Ó, Mesejo P, Ibáñez O (2021) Automatic segmentation of skeletal structures in X-ray images using deep learning for comparative radiography. Forensic Imaging 26:200458. 10.1016/j.fri.2021.200458

[76] Ferrell MJ, Schultz JJ, Adams DM (2026) Combining morphological traits and measurements of the skull for osteological sex estimation using random forest modeling. J Forensic Sci 71:668–682. 10.1111/1556-4029.70241 41320966 10.1111/1556-4029.70241

[77] Ferrell MJ, Schultz JJ, Adams DM (2025) Decision trees for combining morphological traits and measurements of the skull for osteological sex estimation. J Forensic Sci 70:1653–1669. 10.1111/1556-4029.70123 40616150 10.1111/1556-4029.70123

[78] Ferrell MJ, Schultz JJ, Adams DM (2025) The application of decision trees for estimating osteological sex from common measurements of the skull. J Forensic Sci 70:854–867. 10.1111/1556-4029.70031 40135464 10.1111/1556-4029.70031

[79] Fan F, Ke W, Wu W et al (2020) Automatic human identification from panoramic dental radiographs using the convolutional neural network. Forensic Sci Int 314:110416. 10.1016/j.forsciint.2020.110416 32721824 10.1016/j.forsciint.2020.110416

[80] Etli Y, Asirdizer M, Hekimoglu Y, Keskin S, Yavuz A (2019) Sex estimation from sacrum and coccyx with discriminant analyses and neural networks in an equally distributed population by age and sex. Forensic Sci Int 303:109955. 10.1016/j.forsciint.2019.109955 31541936 10.1016/j.forsciint.2019.109955

[81] Epain M, Valette S, Zou K et al (2024) Sex estimation from coxal bones using deep learning in a population balanced by sex and age. Int J Legal Med 138:2617–2623. 10.1007/s00414-024-03268-2 38862820 10.1007/s00414-024-03268-2

[82] Dinkar AD, Sambyal SS (2012) Person identification in ethnic Indian Goans using ear biometrics and neural networks. Forensic Sci Int 223:373.e1-373.e13. 10.1016/j.forsciint.2012.08.032 22980142 10.1016/j.forsciint.2012.08.032

[83] Demir U, Etli Y, Hekimoglu Y et al (2022) Sex estimation from the clavicle using 3D reconstruction, discriminant analyses, and neural networks in an Eastern Turkish population. Leg Med Tokyo 56:102043. 10.1016/j.legalmed.2022.102043 35183842 10.1016/j.legalmed.2022.102043

[84] Demet Mutlu G, Asirdizer M, Kartal E et al (2024) Sex estimation from the hyoid bone measurements in an adult Eastern Turkish population using 3D CT images, discriminant function analysis, support vector machines, and artificial neural networks. Leg Med Tokyo 67:102383. 10.1016/j.legalmed.2023.102383 38159420 10.1016/j.legalmed.2023.102383

[85] Çiftçi R, Atik İ, Eken Ö, Aldhahi MI (2025) Deep learning for sex estimation from whole-foot X-rays: benchmarking CNNs for rapid forensic identification. Diagnostics Basel 15:2923. 10.3390/diagnostics15222923 41300947 10.3390/diagnostics15222923 PMC12651919

[86] Ciconelle ACM, da Silva RLB, Kim JH et al (2023) Deep learning for sex determination: analyzing over 200,000 panoramic radiographs. J Forensic Sci 68:2057–2064. 10.1111/1556-4029.15376 37746788 10.1111/1556-4029.15376

[87] Chomean S, Chatthai N, Sangchay N, Kaset C (2025) Enhancing forensic sex identification through AI-based analysis of the foramen magnum. Forensic Sci Int Rep 11:100411. 10.1016/j.fsir.2025.100411

[88] Choi H-R, Siadari TS, Kim J-E et al (2022) Automatic detection of teeth and dental treatment patterns on dental panoramic radiographs using deep neural networks. Forensic Sci Res 7:456–466. 10.1080/20961790.2022.2034714 36353329 10.1080/20961790.2022.2034714 PMC9639521

[89] Çayabatmaz B, Oner Z, Oner S, Seçgin Y (2026) Traditional sex estimation with parameters obtained from the distal end of the humerus: an example of using machine learning algorithms in morphometric studies. Egypt J Forensic Sci 16:4. 10.1186/s41935-025-00504-7

[90] Cavlak N, Çınarer G, Erkoç MF, Kılıç K (2025) Sex estimation with convolutional neural networks using the patella magnetic resonance image slices. Forensic Sci Med Pathol 21:628–639. 10.1007/s12024-025-00943-7 39969760 10.1007/s12024-025-00943-7 PMC12325482

[91] Cavalli F, Lusnig L, Trentin E (2017) Use of pattern recognition and neural networks for non-metric sex diagnosis from lateral shape of calvarium: an innovative model for computer-aided diagnosis in forensic and physical anthropology. Int J Legal Med 131:823–833. 10.1007/s00414-016-1439-8 27571939 10.1007/s00414-016-1439-8

[92] Cao Y, Ma Y, Yang X et al (2022) Use of deep learning in forensic sex estimation of virtual pelvic models from the Han population. Forensic Sci Res 7:540–549. 10.1080/20961790.2021.2024369 36353321 10.1080/20961790.2021.2024369 PMC9639534

[93] Cao Y, Ma Y, Vieira DN et al (2021) A potential method for sex estimation of human skeletons using deep learning and three-dimensional surface scanning. Int J Legal Med 135:2409–2421. 10.1007/s00414-021-02675-z 34459973 10.1007/s00414-021-02675-z

[94] Bu W-Q, Guo Y-X, Zhang D et al (2023) Automatic sex estimation using deep convolutional neural network based on orthopantomogram images. Forensic Sci Int 348:111704. 10.1016/j.forsciint.2023.111704 37094502 10.1016/j.forsciint.2023.111704

[95] Blanc M, Knecht S, Nguyen K et al (2025) Sexual dimorphism of the humerus bones in a French sample: comparison of several statistical models including machine learning models. Int J Legal Med 139:1395–1408. 10.1007/s00414-025-03417-1 39856332 10.1007/s00414-025-03417-1 PMC12003549

[96] Bidmos MA, Olateju OI, Latiff S et al (2023) Machine learning and discriminant function analysis in the formulation of generic models for sex prediction using patella measurements. Int J Legal Med 137:471–485. 10.1007/s00414-022-02899-7 36205796 10.1007/s00414-022-02899-7 PMC9902304

[97] Bewes J, Low A, Morphett A et al (2019) Artificial intelligence for sex determination of skeletal remains: application of a deep learning artificial neural network to human skulls. J Forensic Leg Med 62:40–43. 10.1016/j.jflm.2019.01.004 30639854 10.1016/j.jflm.2019.01.004

[98] Bertsatos A, Chovalopoulou M-E, Brůžek J, Bejdová Š (2020) Advanced procedures for skull sex estimation using sexually dimorphic morphometric features. Int J Legal Med 134:1927–1937. 10.1007/s00414-020-02334-9 32504147 10.1007/s00414-020-02334-9

[99] Bakan HU, Yetim EE, Ilhanli N et al (2025) Sex estimation from the sternum in Turkish population using various machine learning methods and deep neural networks. Egypt J Radiol Nucl Med 56:83. 10.1186/s43055-025-01583-1

[100] Sathya B, Neelaveni R (2020) Transfer learning based automatic human identification using dental traits- an aid to forensic odontology. J Forensic Leg Med 76:102066. 10.1016/j.jflm.2020.102066 33032205 10.1016/j.jflm.2020.102066

[101] Donato L, Cecchi R, Dagoli S et al (2023) Facial age progression: review of scientific literature and value for missing person identification in forensic medicine. J Forensic Leg Med 100:102614. 10.1016/j.jflm.2023.102614 37976962 10.1016/j.jflm.2023.102614

[102] Donato L, Cecchi R, Ubelaker DH et al (2025) Cam deformity of the hip and personal identification of unidentified remains: two case reports of forensic anthropological interest. Can Soc Forensic Sci J 58:140–149. 10.1080/00085030.2025.2536417

[103] Donato L, Cucurachi N, Ubelaker DH et al (2025) The study of the skeletal remains of the 16th century Italian commander Alessandro Farnese and his wife, Maria D’Aviz. Int J Osteoarchaeol 35:e3384. 10.1002/oa.3384

[104] Donato L, Ubelaker DH, Cecchi R et al (2025) Recovery and study of human remains at the Chapel of S. Domenico – Al-Tahira Cathedral, Iraq: medico-legal and forensic anthropological investigations. Torture 35:26–37. 10.7146/torture.v35i1.150054 10.7146/torture.v35i1.150054 40434701

[105] Donato L, Cecchi R, Ubelaker DH et al (2025) The age progression technique: study and evaluation of the results. Legal Med 74:102611. 10.1016/j.legalmed.2025.102611 40107076 10.1016/j.legalmed.2025.102611

[106] Donato L, Treglia M, Cecchi R et al (2026) Forensic age progression for missing person investigations: a pilot evaluation of recognition accuracy. Legal Med 82:102808. 10.1016/j.legalmed.2026.102808 41687395 10.1016/j.legalmed.2026.102808

[107] Donato L, Treglia M, Ubelaker DH et al (2025) Testing age progression technique: evaluation of reliability and accuracy. Legal Med 79:102731. 10.1016/j.legalmed.2025.102731 41075534 10.1016/j.legalmed.2025.102731

[108] Donato L, Ubelaker DH, Bugelli V et al (2024) Facial growth parameters in Down syndrome: review of the literature and forensic application for missing persons age progression. J Forensic Leg Med 107:102756. 10.1016/j.jflm.2024.102756 39357325 10.1016/j.jflm.2024.102756

[109] Donato L, Ubelaker DH, Bugelli V et al (2025) Forensic age progression application in a morphological study of an Italian family: a case report. Leg Med Tokyo 74:102601. 10.1016/j.legalmed.2025.102601 10.1016/j.legalmed.2025.102601 40015186

[110] Donato L, Ubelaker DH, Marsella L et al (2024) Father figure: study of the age progression process from old pictures and its value in forensic sciences. Legal Med 68:102421. 10.1016/j.legalmed.2024.102421 38401334 10.1016/j.legalmed.2024.102421

[111] Donato L, Ubelaker DH, Marsella LT et al (2024) The forensic imaging technique of age progression used within missing people cases: the Italian Missing Children Association realizing age progression for Missing Child Kenya. Sci Justice 64:210–215. 10.1016/j.scijus.2024.02.002 38431378 10.1016/j.scijus.2024.02.002

[112] Donato L, Ubelaker DH, Marsella LT et al (2025) Applications of forensic anthropology methodology: accuracy of virtual face reproductions performed on the Tenchini Collection. Forensic Sci Med Pathol 21:203–209. 10.1007/s12024-024-00839-y 38795264 10.1007/s12024-024-00839-y

[113] Donato L, Ubelaker DH, Marsella LT et al (2026) The age progression technique to support Missing Children Argentina: a case report. Med Sci Law 66:174–179. 10.1177/00258024251380953 40971618 10.1177/00258024251380953

[114] Cecchi R, Calabrò F, Camatti J et al (2026) Artificial intelligence in healthcare: proposal for a new medico-legal methodology in medical liability. Leg Med 80:102764. 10.1016/j.legalmed.2025.102764 10.1016/j.legalmed.2025.102764 41401747

