# Supplementary material for: Artificial intelligence in forensic science: a systematic review. Part I: personal identification
Source: Int J Legal Med. 2026 Jun 4;140(5):3317–29. doi: 10.1007/s00414-026-03855-5 (PMC13499856; doi:10.1007/s00414-026-03855-5)
Supplement: Supplementary file 1 [file 414_2026_3855_MOESM1_ESM.docx]

**Supplementary Appendix 1**

**Database search strategies**

**PubMed (MEDLINE)**

Search string:

(("artificial intelligence" OR "machine learning" OR "deep learning"

OR "neural network*" OR "convolutional neural network*" OR CNN

OR "transformer model*" OR "computer vision" OR "data-driven")

AND

("forensic" OR "forensic science" OR "forensic medicine"

OR "forensic anthropology" OR "forensic odontology" OR medicolegal)

AND

("personal identification" OR "human identification"

OR "forensic identification" OR "dental identification"

OR "dental radiograph*" OR "panoramic radiograph*"

OR "facial recognition" OR "face recognition"

OR "sex estimation" OR "sex determination"

OR "ancestry estimation" OR "population affinity"

OR "biological profile"))

**Scopus**

Search string:

( "artificial intelligence" OR "machine learning" OR "deep learning" OR "neural network" OR "CNN" OR "computer vision" OR "transformer" ) AND ( forensic OR medicolegal ) AND ( "personal identification" OR "human identification" OR "dental identification" OR "face recognition" OR "sex estimation" OR "ancestry estimation" OR "population affinity" )

**Supplementary Table 1**

Characteristics of the studies included in the systematic review.

Abbreviations: AdaBoost = Adaptive Boosting; AI = Artificial Intelligence; ANN = Artificial Neural Network; AUC = Area Under the Curve; CBCT = Cone-Beam Computed Tomography; CNN = Convolutional Neural Network; CT = Computed Tomography; DENT-net = Dental Network; DFA = Discriminant Function Analysis; DL = Deep Learning; DNN = Deep Neural Network; DT = Decision Tree; DVAE = Disentangled Variational Autoencoder; EER = Equal Error Rate; EFD = Elliptic Fourier Descriptors; F1-score = Harmonic Mean of Precision and Recall; GB = Gradient Boosting; GLM = Generalized Linear Model; GoogLeNet = Google Convolutional Neural Network Architecture; GSN = Greater Sciatic Notch; HOG = Histogram of Oriented Gradients; IoU = Intersection over Union; JRIP = Repeated Incremental Pruning to Produce Error Reduction; KNN = k-Nearest Neighbors; L4 = Fourth Lumbar Vertebra; LDA = Linear Discriminant Analysis; LeNet5 = LeCun Neural Network Architecture; LR = Logistic Regression; MAE = Mean Absolute Error; MCC = Matthews Correlation Coefficient; MDCT = Multidetector Computed Tomography; ML = Machine Learning; MLP = Multilayer Perceptron; MRI = Magnetic Resonance Imaging; NB = Naive Bayes; OPG = Orthopantomogram; PCA = Principal Component Analysis; PHN = Probabilistic Hybrid Network; PLR = Penalized Logistic Regression; PLS = Partial Least Squares; PMCT = Post-Mortem Computed Tomography; pQCT = Peripheral Quantitative Computed Tomography; PR-AUC = Precision–Recall Area Under the Curve; QDA = Quadratic Discriminant Analysis; QPU = Quantum Processing Unit; RDA = Regularized Discriminant Analysis; ResNet = Residual Neural Network; RF = Random Forest; ROC = Receiver Operating Characteristic; SCED = Son-Child Ear Dataset; SHAP = Shapley Additive Explanations; SLR = Score-Based Likelihood Ratio; STR = Short Tandem Repeat; SVC = Support Vector Classifier; SVM = Support Vector Machine; TL = Transfer Learning; TrC = Triticeal Cartilage; U-Net = Convolutional Neural Network Architecture for Image Segmentation; VGG16 = Visual Geometry Group 16-Layer Network; XGBoost = Extreme Gradient Boosting; YOLOv5 = You Only Look Once Version 5.

| **Ref.** | **Study** | **Population / Country** | **Sample** | **Data type** | **Model** | **Task** | **Performance** |
| --- | --- | --- | --- | --- | --- | --- | --- |
| 12 | Zhang et al., 2025 | China | 220 volunteers (1100 fingerprint images) | Fingerprints | CNN | Sex estimation | Accuracy 95%, AUC 0.983 |
| 13 | Wen et al., 2022 | China | 732 patients | CT sphenoid sinus | CNN | Human identification | Rank-1 93.94%, Rank-5 99.24% |
| 14 | Warrier & San-Millán, 2025 | Iberian population | 668 skeletal remains | Acetabulum measurements | ML classifiers (SVC, DTC, KNN, ANN) | Sex estimation | Accuracy up to 89.85% |
| 15 | Wang et al., 2026 | China | 523 subjects (SCED dataset) + 486 (CNKE dataset) | Ear biometric images | Siamese network + VGG16-Transformer | Kinship verification | Accuracy 93.53%, AUC 0.98 |
| 16 | Verma et al., 2021 | Public image datasets | 2000 images | Facial landmarks | Logistic regression / CNN | Sex estimation | AUC 0.94 |
| 17 | Tyagi et al., 2021 | India | 293 hyoid bones | Skeletal morphometrics | XGBoost, SVM, RF, KNN | Sex estimation | Accuracy 83%, AUC 0.89 |
| 18 | Triantafyllou et al., 2024 | Greece | 92 skulls | Orbital measurements | Random Forest | Sex estimation | Accuracy 0.68, AUC 0.72 |
| 19 | Torimitsu et al., 2024 | Japan / Australia | 455 CT scans | Cranial CT measurements | Random Forest, SVM | Ancestry estimation | Accuracy up to 97.1% |
| 20 | Torimitsu et al., 2024 | Japan / Australia | 365 CT scans | Pelvic CT measurements | Random Forest, SVM | Population affinity | Accuracy >90% (SVM sex-specific) |
| 21 | Torimitsu et al., 2024 | Japan / Australia | 627 CT scans | Femoral CT measurements | Random Forest, SVM | Population affinity | Accuracy 71–86% |
| 22 | Torimitsu et al., 2025 | Japan / Malaysia | 434 CT scans (252 Japanese, 182 Malay) | Cranial CT measurements | Random Forest, SVM | Population affinity estimation | Accuracy 88% (RFM), 94.5% (SVM) |
| 23 | Toneva et al., 2020 | Bulgaria | 393 CT scans | Cranial morphometric measurements | JRIP, Ridor, J48 decision tree | Sex estimation | Accuracy up to 91.9% |
| 24 | Spiros & Hefner, 2019 | USA skeletal collections | 254 skeletons | Cranial + postcranial macromorphoscopic traits | ANN, Random Forest, SVM, QDA | Ancestry estimation | Accuracy 88–92% |
| 25 | Sonmez et al., 2026 | Türkiye | 520 calvaria photographs | Skull images | ResNet50 + SVM / KNN | Sex estimation | Accuracy up to 96.77% |
| 26 | Sonmez et al., 2025 | Türkiye | 137 autopsy cases (209 TrC samples) | Triticeal cartilage morphometrics | MLP, RF, SVM, KNN | Sex and stature estimation | Sex accuracy 97% (MLP) |
| 27 | Siino & Sears, 2020 | Simulated forensic genetic datasets | 9,204 pedigrees (9,298 unknown profiles) | STR genetic profiles | Gradient-descent logistic regression cascade AI | Kinship identification / pedigree matching | Improved sensitivity and specificity compared to human interpretation |
| 28 | Schroder et al., 2025 | Brazil | 470 patients | Lateral cephalometric radiographs (sella turcica parameters) | LR, SVM, KNN, MLP, RF, AdaBoost, Gradient Boosting | Sex estimation | AUC ≈ 0.85; precision ≈ 0.77 |
| 29 | Sasani et al., 2024 | Turkey | 200 CT scans | Mastoid triangle measurements + mastoid air cell volume | ANN, SVM, KNN, Gaussian NB | Sex estimation | Accuracy up to 88.5% (ML models) |
| 30 | Santos Mota et al., 2025 | Brazil | 241 skulls | Cranial morphometric measurements | Artificial Neural Network | Sex estimation | High accuracy using reduced measurement set (3 variables outperforming larger models) |
| 31 | Sanil et al., 2023 | India / mixed datasets | Facial image datasets | 2D facial images reconstructed into 3D meshes (468 landmarks) | XGBoost, RF, SVM, AdaBoost, NB, LR | Facial recognition / identification | Best accuracy 78% (XGBoost) |
| 32 | Salmanipour et al., 2023 | Iranian population | 199 individuals (118 M / 81 F) | Skull CT measurements | Gradient Boosting Decision Trees, Logistic Regression | Sex estimation | Gradient Boosting accuracy 0.89; AUC 0.94 |
| 33 | Sagingalieva et al., 2025 | Mixed anthropological dataset | 1400 skull scanograms | Calvarium shape descriptors (Fourier features from MDCT scanograms) | Hybrid Quantum Neural Networks (PHN, HQCNN), CNN, MLP | Sex estimation | Best hybrid model accuracy ≈ 90.7% on QPU |
| 34 | Ray et al., 2025 | Turkish population | 300 individuals (150 M / 150 F) | Talocrural joint radiographs (X-ray morphometric variables) | LR, DT, KNN, LDA, NB, Random Forest | Sex estimation | Accuracy 82–92% (highest with Random Forest) |
| 35 | Porto et al., 2020 | Brazilian population | 18,000 facial images | Photo-anthropometric facial indices (208 PAIs) | Artificial Neural Network (MLP) | Sex and age estimation | Sex estimation F1 > 0.85 (age-group classification F1 > 0.93) |
| 36 | Pichetpan et al., 2023 | Thai population | 200 clavicle pairs | Clavicle photographs | CNN (GoogLeNet) | Sex estimation | Accuracy up to 95% (validation set) |
| 37 | Pereira et al., 2025 | Portuguese population | 1050 orthopantomograms | Dental panoramic radiographs (OPG) | CNN (VGG16) | Sex estimation | Accuracy ≈ 89%, F1-score ≈ 0.89 |
| 38 | Palmela Pereira et al., 2025 | Portuguese population | 1235 orthopantomograms from 1050 patients | Dental OPG radiographs | CNN (VGG16 transfer learning) | Age classification and positive identification | Overall accuracy ≈ 85% |
| 39 | Oura et al., 2023 | Terry Anatomical Collection (USA) | 117 L4 vertebrae scans | pQCT slice of lumbar vertebra | Deep learning (LeNet5-based architecture) | Sex estimation | Accuracy 86.4% |
| 40 | Parlak et al., 2025 | Turkish population | 442 CT scans | Piriform aperture CT measurements | Ensemble ML (ANN, SVM, RF, KNN etc.) | Sex estimation | Accuracy up to 85.7% |
| 41 | Park et al., 2024 | South Korean population | 13,200 panoramic radiographs | Dental panoramic radiographs | Multi-task DL network (ForensicNet, EfficientNet-B3 backbone) | Sex and age estimation | Sex accuracy 0.992; MAE age ≈ 2.93 years |
| 42 | Oura et al., 2023 | Terry Anatomical Collection (USA) | 199 knee radiographs from 100 skeletons | Knee joint radiographs | Deep learning (MhNet architecture) | Sex estimation | Accuracy 90.3% |
| 43 | Ortega et al., 2021 | Spanish population (Granada infant skeletal collection) | 135 ilium photographs (infants) | 2D bone images (ilium) | Deep learning (VGG16, ResNet50) + ML (SVM, RF, AdaBoost) | Sex estimation | DL accuracy ≈ 59% (expert 61%) |
| 44 | Oner et al., 2019 | Turkish population | 422 thoracic CT scans | Sternum measurements from CT | Artificial Neural Network (multilayer perceptron) | Sex estimation | Accuracy 0.906 (Sens 0.91; Spec 0.90) |
| 45 | Oliva et al., 2022 | Italian population | 100 dental casts (premolars) | 3D dental scans | ANN + Geometric Morphometrics | Sex estimation | Test accuracy ≈ 0.80 |
| 46 | Nonthasaen et al., 2023 | Thai population | 1173 hand radiographs | Hand X-ray images | CNN (InceptionResNetV2 best model) | Sex estimation | Accuracy 87.5%; F1-score 86.9% |
| 47 | Nogueira et al., 2023 | French population | 78 radii (36 M, 42 F) | Radius bone measurements | ML + statistical models (LDA, FDA, RDA, PLR, RF, SVM) | Sex estimation | Accuracy up to 97% |
| 48 | Navega et al., 2015 | Portuguese population | 300 individuals (training) + 60 test | Tarsal bone measurements | ML (ANN, classification trees) + DFA, logistic regression | Sex estimation | Accuracy ≈ 88.3% |
| 49 | Navega et al., 2015 (AncesTrees) | Multi-population dataset (Howells’ series) | 1,734 skulls | Craniometric variables | Random Forest | Ancestry estimation | Accuracy up to 93.8% |
| 50 | Musilová et al., 2019 | Czech + French populations | 208 CT cranial scans | 3D exocranial meshes | PCA + Support Vector Machine | Sex estimation / population affinity | Accuracy up to 96.2% |
| 51 | Monum et al., 2024 | Japanese population | 201 PMCT scans | 3D CT rib measurements | ML algorithms (LR, RF, SVM, ANN, etc.) | Sex estimation | Best accuracy 83.6% |
| 52 | Malatong et al., 2023 | Thai population | 220 individuals (1100 lumbar vertebrae images) | Lumbar vertebra photographs | Deep learning (GoogLeNet CNN) + morphometric methods | Sex estimation | DL accuracy up to 92.5% |
| 53 | Maier et al., 2015 | USA skeletal collections | 376 individuals | 3D digitized palate shape data | Shape-matching algorithms + machine learning | Ancestry estimation | Accuracy ≈ 58% using palate shape classification |
| 54 | Machado et al., 2024 | Brazilian population | 54 CBCT scans (10 external test scans) | Craniofacial CBCT measurements | Logistic Regression, Decision Trees, Random Forest, ANN | Sex estimation | Accuracy >80% |
| 55 | Li et al., 2023 | Chinese dataset | Large facial image datasets | Facial recognition features extracted by CNN | Deep learning facial recognition + score-based likelihood ratio | Facial identification | High SLR values supporting forensic facial comparison |
| 56 | Li et al., 2022 | Chinese Han population | 2122 pelvic radiographs | Proximal femur X-ray images | Deep CNN (YOLOv5 detection + ResNet classification) | Sex estimation | Accuracy 94.6% (Chinese test dataset) |
| 57 | Kocamis et al., 2025 | Turkish population | 152 CT scans (77 F / 75 M) | Sacroiliac joint morphometric measurements from CT | SVM, Decision Tree, Logistic Regression | Sex estimation | Best accuracy ≈ 88% (SVM) |
| 58 | Knecht et al., 2026 | Multi-collection skeletal dataset | 2969 individuals | Long bone osteometric measurements | ML classifiers (LDA, RF, SVM, GB, XGB etc.) | Sex estimation | Accuracy up to 93% |
| 59 | Knecht et al., 2025 | Multi-collection skeletal dataset | 2969 individuals | Long bone measurements | Interpretable ML models (LDA, RF, SVM, XGB + SHAP) | Sex estimation | Accuracy up to 95.2% |
| 60 | Knecht et al., 2024 | Italian population | 180 skeletons + 21 validation cases | Patella morphometric measurements | ML classifiers (LR, SVM, RF, XGB etc.) | Sex estimation | Accuracy 0.85–0.95 depending on model |
| 61 | Kuha et al., 2024 | South African population | 193 mandibles | Mandible photographs | Deep learning neural networks | Sex estimation | Male accuracy 100%, female 76.9% |
| 62 | Knecht et al., 2023 | Multi-collection skeletal dataset (France/USA collections) | 2141 individuals | Long bone morphometric measurements (humerus, radius, femur, tibia) | LDA, PLR, Random Forest, SVM, ANN | Sex estimation | Accuracy 90–92% using combined bones |
| 63 | Knecht et al., 2021 | French population | 60 coxal bones | Greater sciatic notch morphometric and morphological variables | LDA, RDA, PLR, FDA, SVM, ANN | Sex estimation | Accuracy 0.92–0.97 depending on variables |
| 64 | Kim et al., 2025 | Korea / Japan multi-institution dataset | 254 CT scout images | Post-mortem CT scout images (metal implants) | RetinaNet, Faster R-CNN | Implant detection for personal identification | Improved detection with reduced false positives |
| 65 | Kim et al., 2025 | Korean dental dataset | 2067 orthopantomograms | Dental panoramic radiographs | CNN (multi-task deep learning with VGG backbone) | Sex and age estimation | Sex accuracy 90.2%; MAE age 3.43 years |
| 66 | Kim et al., 2024 | Japanese dataset | 110 PMCT cases + clinical CXR images | Chest radiographs / pseudo-2D X-ray bone projections | U-Net deep learning | Bone extraction for radiographic identification | High Dice/IoU segmentation performance |
| 67 | Kartal et al., 2022 | Turkish population | 720 CT scans (360 M / 360 F) | Foramen magnum measurements from cranial CT | Artificial Neural Network + discriminant analysis | Sex estimation | ANN accuracy 88.2%; discriminant analysis 86.7% |
| 68 | Karim et al., 2026 | Iraqi population | 200 subjects (100 M / 100 F) | Digital dental impressions (maxillary anterior teeth) | Machine learning classifiers (SVC best model) | Sex estimation | Accuracy 94.5% (SVC) |
| 69 | Intasuwan et al., 2022 | Thai skeletal collection | 250 coxae | Greater sciatic notch photographs | Deep learning CNN | Sex estimation | DL accuracy 91.95% (vs 90.3% morphometry) |
| 70 | Imaizumi et al., 2025 | Japanese PMCT dataset | 240 skulls | 3D skull shapes from PMCT | PCA/PLS dimensionality reduction + SVM | Sex estimation | Accuracy >90% in several skull regions |
| 71 | Imaizumi et al., 2020 | Japanese PMCT dataset | 100 skulls | 3D skull models reconstructed from PMCT | PCA/PLS + SVM | Sex estimation | Up to 100% accuracy in some skull parts |
| 72 | Huang et al., 2025 | Chinese volunteers | 10 individuals sampled longitudinally | Skin microbiome (16S rRNA sequencing + bacterial SNP profiles) | Random Forest, XGBoost, SVM | Individual identification | >97.5% accuracy using microbial SNP profiles |
| 73 | Attia et al., 2022 | Turkish population | 300 individuals | Femoral osteometric measurements from CT | LDA, SVM, Logistic Regression, Boosted GLM (ABC classification approach) | Sex estimation | Accuracy up to 96–100% in testing sample |
| 74 | Guo et al., 2024 | Chinese Han population | 477 CBCT scans | Maxillary sinus measurements | Logistic, KNN, SVM, Random Forest | Sex estimation | RF best model; overall accuracy ≈77.8% (up to 88.5% with age stratification) |
| 75 | Gomez et al., 2021 | Radiographic datasets (Spain hospital + JSRT dataset) | 200 radiographs for training | X-ray radiographs (clavicles and frontal sinuses) | CNN deep learning segmentation (X-Net+) | Skeletal segmentation for comparative radiography identification | Dice score 0.939 (clavicles) and 0.823 (frontal sinuses) |
| 76 | Ferrell et al., 2025 | US skeletal collections | 403 individuals | Skull morphological traits + cranial measurements | Random Forest | Sex estimation | Classification accuracy >80% in multiple models |
| 77 | Ferrell et al., 2025 (decision trees) | US skeletal collections | 403 individuals | Skull morphological traits + cranial measurements | Decision tree ML models | Sex estimation | Accuracy 87.9–100% depending on skull region |
| 78 | Ferrell et al., 2024 | US skeletal collections | 403 individuals | Skull measurements (21 craniometric variables) | Decision tree classifiers | Sex estimation | Accuracy up to 95% in cranium models |
| 79 | Fan et al., 2020 | Chinese dental dataset | 15,868 panoramic dental radiographs from 6,473 individuals | Panoramic dental radiographs | CNN (DENT-net) | Human identification | Rank-1 accuracy 85.16%; Rank-5 accuracy 97.74% |
| 80 | Etli et al., 2019 | Turkish population | 480 CT scans | Sacrum and coccyx morphometric measurements | Discriminant analysis + multilayer perceptron neural network | Sex estimation | Neural network accuracy 86.3% |
| 81 | Epain et al., 2024 | French population | 580 CT scans | 3D pelvic (coxal bone) CT reconstructions | Deep learning (DVAE + Crecon classifier) | Sex estimation | Accuracy up to 99.8% |
| 82 | Dinkar & Sambyal, 2012 | Indian (Goan) population | 400 individuals (800 ears) | Digital ear photographs | Neural networks + weighted scoring system | Biometric identification | Recognition rate 94%, EER 0.225 |
| 83 | Demir et al., 2022 | Eastern Turkish population | 360 CT scans | Clavicle morphometric measurements | Discriminant analysis + multilayer perceptron neural network | Sex estimation | Neural network accuracy 91.4% |
| 84 | Demet Mutlu et al., 2024 | Eastern Turkish population | 240 CT scans | Hyoid bone measurements | DFA, SVM, Artificial Neural Networks | Sex estimation | ANN accuracy 95.4% |
| 85 | Çiftçi et al., 2025 | Adult hospital population | 471 foot radiographs | Whole-foot X-ray images | CNN architectures (AlexNet, ResNet, InceptionV3) | Sex estimation | Best accuracy 97.1% (InceptionV3) |
| 86 | Ciconelle et al., 2023 | Brazilian population | 207,946 panoramic radiographs | Dental panoramic X-rays | CNN and ResNet deep learning models | Sex determination | CNN accuracy 95.02%, AUC >96% |
| 87 | Chomean et al., 2025 | Thai skeletal collection | 600 dry skull images (300 M / 300 F), augmented to 2280 images | Photographs of skull base (foramen magnum) | CNN-based object detection and instance segmentation | Sex estimation | Object detection accuracy 65.68% (precision up to 95%) |
| 88 | Choi et al., 2022 | Korean dental radiology dataset | 1,638 dental panoramic radiographs | Dental panoramic radiographs | EfficientDet-D3 CNN | Automated detection of teeth and dental treatment patterns for identification | Average precision up to 99.1% for natural teeth detection |
| 89 | Çayabatmaz et al., 2026 | Turkish population | 310 CT scans (155 M / 155 F) | Distal humerus morphometric parameters from CT | Multiple ML algorithms (LDA, QDA, RF, k-NN, DT, etc.) | Sex estimation | Accuracy range 0.87–0.97 depending on algorithm |
| 90 | Cavlak et al., 2025 | Turkish hospital population | 6,710 patella MRI slices from 696 patients | Patella MRI images | CNN architectures (EfficientNetB3, ResNet50, VGG16, DenseNet121) | Sex estimation | Best accuracy 88.88% (ResNet50) |
| 91 | Cavalli et al., 2017 | Italian Caucasian population | 1700 lateral CT scanograms (850 M, 850 F) | Lateral calvarial shape from CT scout views | Pattern recognition + artificial neural networks | Sex estimation | Accuracy >80% (multiple classifier system) |
| 92 | Cao et al., 2022 | Han Chinese population | 862 pelvic CT scans (437 F, 425 M) | CT-based 3D virtual hemi-pelvis images | Deep learning CNN (GoogLeNet Inception-V4, transfer learning) | Sex estimation from pelvic regions (ventral pubis, dorsal pubis, GSN, pelvic inlet, ischium, acetabulum) | Accuracy up to 100%, AUC up to 1.00; CNN outperformed expert anthropologists |
| 93 | Cao et al., 2021 | Han Chinese population | 1000 pelvic CT scans for model development + 105 real pelvises scanned in 3D | CT-based virtual pelvic images and handheld 3D surface scans | Deep learning CNN (GoogLeNet Inception-V4) | Sex estimation from ventral pubis, dorsal pubis, and greater sciatic notch | CT testing accuracy: 98.0% |
| 94 | Bu et al., 2023 | Northern Han Chinese population | 10,703 orthopantomograms | Orthopantomogram (OPG) images | Deep CNN (CoAtNet/TResNet-based model) | Sex estimation | Accuracy 90.97% in adults; 82.64% in minors; overall AUC 90.64% |
| 95 | Blanc et al., 2025 | French population | 98 left humeri (training sample n = 60; test sample n = 38) | Humerus osteometric measurements | Statistical and machine learning models (LDA, RDA, PLR, FDA, SVM, ANN, RF) | Sex estimation | Cross-validation accuracy 92–98%; up to 98–100% after variable selection; best models: PLR, RF, and LDA |
| 96 | Bidmos et al., 2023 | South African populations (SAAD, SAED, MASA) | 260 patellae (130 M, 130 F) | Osteometric measurements of the patella | Discriminant Function Analysis and Machine Learning (RF, XGBoost, AdaBoost, KNN, Gradient Boosting, stacking model) | Sex estimation | DFA accuracy 81.9–84.2%; ML accuracy up to 89.6%; stacking model accuracy 90.8% (AUC 92.65%) |
| 97 | Bewes et al., 2019 | Australian hospital population | 1000 skull CT scans (500 M, 500 F); test set n = 100 | 3D skull reconstructions from CT scans (2D lateral images) | Deep learning convolutional neural network (GoogLeNet, transfer learning) | Sex estimation | Overall accuracy 95%; Matthews correlation coefficient 0.90 |
| 98 | Bertsatos et al., 2020 | Czech and Greek populations | 324 crania (170 Czech CT-derived models; 154 Greek skeletal models) | 3D cranial models with extracted morphometric features (glabella, supraorbital ridge, occipital protuberance, mastoid process) | Computer vision and supervised machine learning (HOG + SVM; EFD + LDA; combined multi-feature classifier) | Sex estimation | Population-specific accuracy 78.5–96.7%; population-generic accuracy 71.7–90.8%; combined multi-feature accuracy >91% |
| 99 | Bakan et al., 2025 | Turkish population | 485 thorax CT cases (248 M, 237 F) | Sternum CT measurements and sagittal/coronal sternum images | Machine learning models (KNN, RF, XGBoost, Naive Bayes, Logistic Regression, LDA) and Deep Neural Network (DNN) | Sex estimation | Best model: LDA (AUC 0.961, accuracy 0.920); DNN AUC 0.937, accuracy 0.878 |
| 100 | B & R, 2020 | Indian population | 106 panoramic radiographs (3159 teeth) | Dental panoramic radiographs | Transfer learning with AlexNet (three-stage CNN pipeline) | Human identification using dental traits | Tooth location accuracy 100%; tooth class accuracy 95–95.5%; tooth numbering accuracy 86.5–94.7%; top-1 identification 95.34%, top-5 identification 100% |
